# A Novel Complex-Coefficient In-Band Interference Suppression Algorithm for Cognitive Ultra-Wide Band Wireless Sensors Networks

**DOI:** 10.3390/s17061206

**Published:** 2017-05-25

**Authors:** Hailiang Xiong, Wensheng Zhang, Hongji Xu, Zhengfeng Du, Huaibin Tang, Jing Li

**Affiliations:** 1 School of Information Science and Engineering, Shandong University, Jinan 250100, China; hongjixu@sdu.edu.cn (H.X.); zfdu@sdu.edu.cn (Z.D.); 2 School of Microelectronics, Shandong University, Jinan 250100, China; tanghuaibin@sdu.edu.cn; 3 Shanghai Institute of Technical Physics, Chinese Academy of Sciences, Shanghai 200083, China; Riejing@126.com

**Keywords:** ultra-wide band, cognitive radio, signal to interference and noise ratio, wireless sensor networks, interference avoiding, notch filter, power spectrum density, time hopping, complex-coefficient, spread spectrum, bit error ratio

## Abstract

With the rapid development of wireless communication systems and electronic techniques, the limited frequency spectrum resources are shared with various wireless devices, leading to a crowded and challenging coexistence circumstance. Cognitive radio (CR) and ultra-wide band (UWB), as sophisticated wireless techniques, have been considered as significant solutions to solve the harmonious coexistence issues. UWB wireless sensors can share the spectrum with primary user (PU) systems without harmful interference. The in-band interference of UWB systems should be considered because such interference can severely affect the transmissions of UWB wireless systems. In order to solve the in-band interference issues for UWB wireless sensor networks (WSN), a novel in-band narrow band interferences (NBIs) elimination scheme is proposed in this paper. The proposed narrow band interferences suppression scheme is based on a novel complex-coefficient adaptive notch filter unit with a single constrained zero-pole pair. Moreover, in order to reduce the computation complexity of the proposed scheme, an adaptive complex-coefficient iterative method based on two-order Taylor series is designed. To cope with multiple narrow band interferences, a linear cascaded high order adaptive filter and a cyclic cascaded high order matrix adaptive filter (CCHOMAF) interference suppression algorithm based on the basic adaptive notch filter unit are also presented. The theoretical analysis and numerical simulation results indicate that the proposed CCHOMAF algorithm can achieve better performance in terms of average bit error rate for UWB WSNs. The proposed in-band NBIs elimination scheme can significantly improve the reception performance of low-cost and low-power UWB wireless systems.

## 1. Introduction

Broadband wireless communications, electronics and sensor networks technologies have evolved dramatically in the past twenty years. The growth tendency of wireless sensor networks and the electronics devices market is expected to continue in the following few decades, as the demand for information services is increasing. With more and more communication system and electronic devices going to wireless, the next generation of wireless sensor networks will be confronted with frequency spectral crowding, and coexistence with the pre-existing wireless devices will be one of the most important challenges [1,2,3,4]. Cognitive radio (CR) techniques [2,5,6] and ultra-wide band (UWB) radio communication techniques are considered as a part of the most important solutions for friendly coexistence. In recent years, ultra-wide band (UWB) wireless communication and sensor network technologies have attracted considerable interests in academia and industry [7,8,9,10], due to their promise to provide precision positioning and reliable communication ability at low cost with extremely low energy consumption [11,12]. Ultra-wide band wireless sensor networks are characterized as the transmitted signals with instantaneous frequency spectral occupancy greater than 500 MHz, or with a fractional bandwidth of more than 20% [13]. UWB radio techniques originally started with the spark-gap transmitter of Hertz and Marconi. However, it was not until the 1990s that the interest was renewed. The pioneering work of developing the concept of time-hopping (TH) impulse-based ultra-wide band radio systems can be found in [8,9]. In recent years, the impulse-based UWB techniques have been considered as a competitive candidate for the low-cost high performance wireless sensor networks.

UWB wireless sensors promise to coexist with other licensed and unlicensed narrow band or broadband wireless signals [14]. To ensure the UWB signal without causing significant interferences to the incumbent systems, a number of very strict regulations are imposed on UWB wireless sensor networks [15]. According to the frequency spectrum regulations of the Federal Communications Commission (FCC), UWB wireless sensors must transmit below specified power levels in order not to cause notable interferences with the pre-existing wireless devices in the same frequency spectrum. The detailed average power spectral density (PSD) limitation of the transmitted UWB radios for the indoor environment and the outdoor environment can be found in [13]. However, from the narrow band interferences’ side point of view, the effects of pre-existing narrow band radios on the UWB wireless sensor networks can still be notable. In some extreme situations, the strong interference signals may jam the reception module of the UWB wireless sensors thoroughly [16,17]. The unifying mathematical framework is developed to characterize the aggregate interference in wireless heterogeneous networks [18,19,20]. The aggregate multiple access interferences and the narrow band interferences are modeled as Gaussian random variables from which the variance is determined [21,22,23]. Even if the bandwidth of narrow band interferences is only a small fraction of the huge frequency spectrum UWB radio occupied, due to their relatively high PSD with respect to the primary ultra-wide band radios, the reception performance of the UWB wireless sensors can be influenced enormously. The work in [24] indicated that the average probability of error (PE) of the ultra-wide band sensors will be observably degraded due to the influence of in-band pre-existing strong narrow band interferences (NBIs). The inherent processing gain of the impulse-based UWB wireless signal can resist the narrow band interferences to a certain level [9,25]. However, on many practical occasions, even the inherent processing gain is insufficient enough to compensate the impacts of in-band pre-existing strong NBIs [26,27]. Consequently, either the UWB wireless sensors network designer needs to consider how to avoid the transmission of the UWB wireless signal over the frequencies of strong narrow band interferences or the UWB receivers need to utilize narrow band interference suppression techniques to elevate the reception performance, the transmitted range, the system capacity and the data rate of the UWB radio devices.

In recent years, the significant progress in UWB wireless communications and electronics has enabled the development of low-cost, low-power, multi-functional sensors nodes that are small in size and communicate untethered in a short range [28,29,30,31]. Compared with traditional narrow band and broadband wireless communication systems, interferences suppression in UWB wireless sensor networks is a more challenging issue due to the rather restricted PSD transmission and the extremely wide frequency band occupied [3,32]. Given the low power consumed and low cost requirements in practical UWB wireless senors applications, at the same time noticing other basic limitations such as low computation complexity in both hardware and software, the in-band narrow band interferences issue should be disposed of more specifically, and some appropriate approaches that are able to deal with narrow band interference need to be developed [33,34,35,36].

A universal consideration for narrow band interferences’ alleviation is avoiding the interferences at the transmitter. The work in [37,38] presented the methods via designing the optimal time-hopping sequences or direct spread spectrum codes with the proper waveform to mitigate the narrow band interferences. If the statistic characteristics of the narrow band interference are known, the narrow band interferences can be avoided by means of adjusting the transmission parameters at the transmitter appropriately, since the impacts of interferences can be directly related to the spectral characteristics of the impulse UWB waveform at the receiver. Once the sub-band of frequency spectrum where narrow band interferences are present can be avoided, the impacts of in-band narrow band interferences on the UWB wireless sensors can be alleviated. A good example for the implementation of the above ideal is designing a proper pulse shape via combination and optimization of a number of the basic Gaussian waveform and the *n*-th order derivative of the Gaussian monocycle [39]. In addition, interference avoiding can be realized by some physical solutions. In [40], a narrow band interference avoidance strategy based on frequency notch antenna design is presented. The basic consideration is to create frequency notches by adding a slight resonant module to the ultra-wide band antenna, which makes it insensitive to some featured frequencies. In the above approach, we should note that the reception performance of the ultra-wide band antenna will deteriorate with the number of notches increasing. Therefore, the idea of the frequency notched antenna is not practical enough at avoiding a number of strong in-band narrow band interferences in the same UWB wireless sensor networks.

Another interference alleviation approach is canceling the interferences at the UWB receiver [41,42,43]. Interference detection and avoiding in the frequency domain are common considerations [44]. In addition, the front end analog frequency domain notch fixed filtering strategy at the ultra-wide band wireless sensor networks seems to have high feasibility. The main deficiencies of the above-mentioned NBI-avoiding algorithms are that they are heavily dependent on the precision of a priori knowledge of the central frequency and the bandwidth of the NBIs and channel state information [45,46]. Without obtaining the exact information on the central frequencies of the interferences, the analog filter will become unavailable for interference canceling. Assuming the integral knowledge about the narrow band interference is known, when there is a set of narrow band interferences and the central frequency or the amplitude of the narrow band interferences is varying, methods utilizing analog notch filters or notch antennas may lose their practicality. In addition, utilizing traditional fixed analog filters in the frequency domain will no doubt increase the computation complexity, size, power consumption and cost of the ultra-wide band wireless sensor networks.

In this article, we present a novel complex-coefficient in-band adaptive NBI-avoiding scheme for time hopping coding division impulse ultra-wide band wireless sensor networks. To simplify the reception processing, a novel adaptive complex-coefficient iteration relationship based on Taylor series of the one order complex value notch filter unit with a single zero-pole pair is derived. Based on our previous works [35,36], to cope with multiple in-band strong NBIs in the same ultra-wide band wireless sensor networks, a complex-coefficient linear cascaded high order adaptive notch filter (ANF) and a novel cyclic cascaded high order matrix adaptive notch filter (CCHOMAF) NBI-elimination algorithm based on the proposed basic ANF units are presented. The proposed method is able to elevate the reception performance of UWB wireless sensor networks, not only in additive white Gaussian noise (AWGN) environments, but also in the ultra-wide band multipath fading channels. Since the proposed interference suppression processing can be executed before signal demodulation, it is also fit for the non-coherent impulse UWB wireless sensors [47]. The theoretical analysis and numerical simulation results indicate that the proposed CCHOMAF algorithm has perfect performance in ultra-wide band wireless sensor networks under strong in-band NBIs, in which an ideal average bit error probability performance versus signal to interference and noise ratio (SINR) enhancement is acquired. Overall, the proposed complex-coefficient adaptive notch filter the in-band NBI-elimination scheme can greatly elevate the immunity against NBIs for the ultra-wide band wireless sensors, which is quite suitable for improving the reception performance of low-cost low-power consumption cognitive ultra-wide band wireless sensor networks.

The remainder of the article is outlined as follows. The time hopping code division pulse position modulation (PPM)-based impulse radio (IR) ultra-wide band wireless signal model is presented in Section 2. The reception performance under the AWGN channel and under the multipath fading propagation channel in the presence of narrow band interferences are discussed and compared in Section 3. The detailed procedure of the basic complex-coefficient adaptive notch filter unit with a single zero-pole pair is developed, and the closed form relationship of the weight coefficient based on Taylor series expansion is derived in Section 4. To cope with multiple in-band narrow band interferences, a linear cascaded high order adaptive notch filter and a novel cyclic cascaded high order adaptive notch filter interference elimination algorithm based on the proposed basic one order adaptive notch filter unit are also considered in Section 4. The numerical results are presented and the reception performances and performance elevated under different channel conditions are discussed in Section 4. Finally, Section 5 draws the conclusions of the article.

## 2. Ultra-Wide Band Senor Networks Signal Model

We consider binary time hopping code division pulse position modulation (PPM) impulse radio ultra-wide band wireless sensor networks with $N_{u}$ users [48], where the transmitted UWB wireless signal with PPM from the *q*-th user can be written as [36]

(1) $$\begin{array}{c} s_{\left[q\right]}\left(t\right)=\sqrt{\frac{E_{\left[q\right]}}{N_{f}}}\sum_{i=0}^{\infty }\Xi_{\left[q\right]}\left(\left\lfloor \frac{i}{N_{f}}\right\rfloor \right)d_{\left[q\right]}\left(i\right)\;p_{\mathrm{tx}}\;\left(t-iT_{f}-c_{\left[q\right]}^{\mathrm{TH}}\left(i\right)T_{c}-\varsigma_{\left[q\right]}\right), \end{array}$$

where $E_{\left[q\right]}$ denotes the average energy per transmitted symbol of the *q*-th user; each symbol consists of $N_{f}$ basic pulses $p_{\mathrm{tx}}\left(t\right)$ with an ultrashort time duration $T_{p}$; the frame period is $T_{f}$ seconds; the number of time slots per frame is $N=T_{f}/T_{c}$; the chip period is $T_{c}$; commonly, the chip period is longer than the impulse duration; $\Xi_{\left[q\right]}\left(\left\lfloor i/N_{f}\right\rfloor \right)$ denotes the information symbol transmitted by the *q*-th ultra-wide band sensor; the index of the symbol is $\left\lfloor i/N_{f}\right\rfloor$ ($\left\lfloor z\right\rfloor$ denotes the integer part of *z*); $d_{\left[q\right]}\left(i\right)$ is binary random variables taking values ±1 with equal probability; and $c_{\left[q\right]}^{\mathrm{TH}}\left(i\right)$ is the time hopping code of the *q*-th user, where $c_{\left[q\right]}^{\mathrm{TH}}\left(i\right)\in \left\{0,1,...,N_{h}-1\right\}$; the timing jitter of the *q*-th user $\varsigma_{\left[q\right]}∼U\left(0,T_{c}\right)$ with U(a,b) denoting the uniform distribution on the interval [a,b].

We choose the impulse wave $p_{\mathrm{tx}}\left(t\right)$ to be the second derivative of a Gaussian monocycle, which can be expressed as:

(2) $$p_{\mathrm{tx}}\left(t\right)=\sqrt{\frac{1}{\sqrt{\pi }\tau_{p}}}\left[1-4\pi {\left(\frac{t}{\tau_{p}}\right)}^{2}\right]\exp\left[-2\pi {\left(\frac{t}{\tau_{p}}\right)}^{2}\right],$$

where $\tau_{p}$ is the normalization duration factor controlling the 3-dB bandwidth.

## 3. Performance Analysis

### 3.1. Analysis for AWGN without Narrow Band Interference

We first consider a simple case, where the ultra-wide band signal is transmitted in the AWGN channel without narrow band interference. The received signal over the AWGN channel with $N_{u}$ ultra-wide band users can be expressed as:

(3) $$\begin{array}{c} r_{a}\left(t\right)=\sum_{q=1}^{N_{u}}\sqrt{\frac{E_{\left[q\right]}}{N_{f}}}\sum_{i=0}^{\infty }\Xi_{\left[q\right]}\left(\left\lfloor \frac{i}{N_{f}}\right\rfloor \right)d_{\left[q\right]}\left(i\right)p_{\mathrm{rx}}\;\left(t-iT_{f}-c_{\left[q\right]}^{\mathrm{TH}}\left(i\right)T_{c}-\tau_{\left[q\right]}\right)+\sigma_{n}^{2}n\left(t\right), \end{array}$$

where $p_{\mathrm{rx}}\left(t\right)$ represents the received ultra-wide band impulse, $\tau_{\left[q\right]}$ denotes the propagation time delay of the *q*-th user and n(t) denotes the normalized white noise.

In AWGN channel, the ultra-wide band wireless receiver commonly consists of a correlator structure or matched filter (MF), where the received hybrid signals are correlated with the local template at the receiver. The local template of the *k*-th information bit for the *p*-th user can be expressed as:

(4) $$\begin{array}{c} s_{\mathrm{temp},[p]}\left(t\right)=\;\;\;\sum_{i=kN_{f}}^{\left(k+1\right)N_{f}-1}\;\;\;d_{\left[p\right]}\left(i\right)p_{\mathrm{rx}}\;\left(t-iT_{f}-c_{\left[p\right]}^{\mathrm{TH}}\left(i\right)T_{c}-\varsigma_{\left[p\right]}\right), \end{array}$$

where the *p*-th user is assumed to be the primary user.

The output of the local correlator for the *p*-th user can be written as:

(5) $$\begin{array}{c} y_{\mathrm{mf}}=\int r_{a}\left(t\right)s_{\mathrm{temp},[p]}\left(t\right)dt=\sqrt{\frac{E_{\left[p\right]}}{N_{f}}}\Xi_{\left[p\right]}\;\;\sum_{i=kN_{f}}^{\left(k+1\right)N_{f}-1}\;\;R_{\mathrm{rx}}\left(\varsigma_{\left[p\right]}\left(i\right)\right)+y_{\mathrm{mai}}+n, \end{array}$$

where $R_{\mathrm{rx}}\left(x\right)=\int_{-\infty }^{+\infty }p_{\mathrm{rx}}\left(t\right)p_{\mathrm{rx}}\left(x-t\right)dt$ is the autocorrelation function of the received ultra-wide band pulse waveform, $y_{\mathrm{mai}}$ denotes the multi address interferences (MAIs) from other ultra-wide band users and *n* denotes the output noise, which can be modeled as a normal distribution $n∼N(0,N_{f}\sigma_{n}^{2})$.

Commonly, the second term MAIs can be rewritten as the sum of the multi-access interference terms from each user:

(6) $$\begin{array}{c} y_{\mathrm{mai}}=\sum_{q=1}^{p-1}\sqrt{\frac{E_{\left[q\right]}}{N_{f}}}y_{\left[q\right]}+\sum_{q=p+1}^{N_{u}}\sqrt{\frac{E_{\left[q\right]}}{N_{f}}}y_{\left[q\right]}, \end{array}$$

where each interference term is in turn the summation of interference to the pulses of the desired user. Considering the symbol synchronous and chip synchronous code division case, $y_{\left[q\right]}$ is asymptotically distributed as [49]:

(7) $$\begin{array}{c} y_{\left[q\right]}∼N(0,\frac{N_{f}}{N}\{E\left\{R_{\mathrm{rx}}^{2}\left(\varsigma_{\left[q\right]}\right)\right\}+E\{R_{\mathrm{rx}}^{2}(T_{c}-|\varsigma_{\left[q\right]}|)\}\}). \end{array}$$

Thus, the unconditional bit error probability under AWGN channel can be expressed approximately as:

(8) $$\begin{array}{c} P_{e}\approx Q\left(\frac{\sqrt{E_{1}}E\left\{R_{\mathrm{rx}}^{2}\left(\varsigma_{\left[p\right]}\right)\right\}}{\sqrt{\frac{1}{N}\left(\sum_{q=1}^{p-1}E_{\left[q\right]}\Upsilon_{\left[q\right]}+\sum_{q=p+1}^{N_{u}}E_{\left[q\right]}\Upsilon_{\left[q\right]}\right)+\sigma_{n}^{2}}}\right), \end{array}$$

where $\Upsilon_{\left[q\right]}=E\left\{R_{\mathrm{rx}}^{2}\left(\varsigma_{\left[q\right]}\right)\right\}+E\{R_{\mathrm{rx}}^{2}(T_{c}-|\varsigma_{\left[q\right]}\left|)\right\}$, $\sigma_{n}^{2}=\mathrm{Var}\left\{R_{\mathrm{rx}}\left(\varsigma_{\left[p\right]}\right)\right\}$, and the *Q*-function Q(z) is defined as:

(9) $$\begin{array}{c} Q\left(z\right)=\frac{1}{\sqrt{2\pi }}\int_{z}^{+\infty }exp\left(-\frac{\Theta^{2}}{2}\right)d\Theta . \end{array}$$

### 3.2. Analysis for Multipath Fading Channel in the Presence of In-Band Narrow Band Interferences

The practical UWB propagation environment is a dense multipath fading channel with the impulse response of the Saleh-Valenzuela (SV) model [12,50,51], which can be expressed in general as:

(10) $$\begin{array}{c} h\left(t\right)=\sum_{l=1}^{L}\sum_{k=1}^{K}\alpha_{k,l}\exp\left(j\phi_{k,l}\right)\delta \left(t-T_{l}-\tau_{k,l}\right), \end{array}$$

where $\alpha_{k,l}$ denotes the coefficient of the *k*-th component in the *l*-th cluster, $T_{l}$ denotes the time delay of the *l*-th cluster, $\tau_{k,l}$ denotes the time delay of the *k*-th MPC relative to the *l*-th cluster arrival time $T_{l}$ and $\phi_{k,l}$ denotes the random phase, which is a uniform distribution random variable.

The distributions of the cluster arrival times $T_{l}$ can be approximated by a basic Poisson process, and the ray arrival times $\tau_{k,l}$ can be given by a hybrid Poisson random process. The power delay profile is exponential within each cluster:

(11) $$E\left\{{\left|\alpha_{k,l}\right|}^{2}\right\}=\Omega_{l}\frac{1}{\gamma_{l}\left[\left(1-\beta \right)\lambda_{1}+\beta \lambda_{2}+1\right]}\exp\left(-\tau_{k,l}/r_{l}\right),$$

where $\Omega_{l}$ denotes the integrated energy of the *l*-th cluster and $\gamma_{l}$ denotes the intra-cluster decay time constant.

Commonly, the cluster decay rates depend linearly on the arrival time of the cluster:

(12) $$r_{l}\propto k_{\gamma }T_{l}+\gamma_{0},$$

where $k_{\gamma }$ describes the increase of the decay constant with delay.

The mean energy of the *l*-th cluster follows in general an exponential decay:

(13) $$10\log\left(\Omega_{l}\right)=10\log\left(\exp\left(-T_{l}/\Gamma \right)\right)+M_{\mathrm{cluster}},$$

where $M_{cluster}$ is a normally-distributed variable with standard deviation $\sigma_{cluster}$ around it.

For the simplicity of the analysis, we use the modified multi-path channels according to (10); then, the channel propagation model can be considered as [50,52]:

(14) $$\begin{array}{c} h_{\mathrm{MP}}\left(t\right)=\sum_{l_{p}=1}^{L_{p}}\alpha_{l_{p}}\delta \left(t-\tau_{l_{p}}\right), \end{array}$$

where we consider $L_{p}$ resolvable multi-path components, $\alpha_{l_{p}}$ denotes the fading coefficient of the $l_{p}$-th multi-path and $\tau_{l_{p}}$ denotes the time delay of the $l_{p}$-th multi-path. Therefore, the received UWB signal through the multi-path channel can be written as:

(15) $$\begin{array}{cc} s^{U}\left(t\right) & =\sum_{q=1}^{N_{u}}s_{\left[q\right]}\left(t\right)⊗h_{\mathrm{MP}}\left(t\right)\\ & =\sum_{q=1}^{N_{u}}\sqrt{\frac{E_{\left[q\right]}}{N_{f}}}\sum_{i=0}^{\infty }\Xi_{\left[q\right]}\left(\left\lfloor \frac{i}{N_{f}}\right\rfloor \right)d_{\left[q\right]}\left(i\right)u_{\left[q\right]}\;\left(t-iT_{f}-c_{\left[q\right]}^{\mathrm{TH}}\left(i\right)T_{c}-\tau_{\left[q\right]}\right), \end{array}$$

where:

(16) $$\begin{array}{c} u_{\left[q\right]}\left(t\right)=\sum_{l_{p}=1}^{L_{p}}\alpha_{\left[q\right],l_{p}}p_{\mathrm{rx}}(t-\tau_{\left[q\right],l_{p}}+\tau_{[q],0}), \end{array}$$

with $p_{\mathrm{rx}}\left(t\right)$ being the received UWB pulse with unit energy.

The impact of interferences in UWB-based heterogeneous wireless sensor networks can be modeled by stochastic geometry, and the signal $s_{m}^{N}\left(t\right)$ transmitted by the *m*-th narrow band interference [53] can be written as:

(17) $$\begin{array}{c} s_{m}^{N}\left(t\right)=\sqrt{2E_{N}}\sum_{n=-\infty }^{\infty }a_{m,n}^{N}g\left(t-nT_{c}^{N}-\tau_{m}\right)\times cos\left(2\pi f_{m}^{N}\left(t-\tau_{m}\right)+\theta_{m,n}^{N}\right), \end{array}$$

where $E_{N}$ denotes the average energy per transmitted symbol, g(t) denotes the normalized ultra-wide band baseband pulse waveform satisfying the Nyquist criterion, $a_{m,n}^{N}$ denotes the *m*-th transmitted baseband symbol of the *m*-th NBI, $f_{m}^{N}$ denotes the central frequency of the *m*-th in-band NBI component and $\theta_{m,n}^{N}$ denotes the random phase.

Commonly, the above reception NBI component can be approximated by a tone with the frequency $f_{m}^{N}$, so the NBIs can be written as:

(18) $$\begin{array}{c} s^{N}\left(t\right)\approx \sqrt{\frac{2E_{N}}{T_{c}^{N}}}\sum_{m=1}^{M}cos\left(2\pi f_{m}^{N}\left(t-\tau_{m}\right)+\theta_{m}\right), \end{array}$$

where $T_{c}^{N}$ denotes the average symbol duration of in-band narrow band interference and $\tau_{m}$ denotes the random time delay of the *m*-th NBI.

The total received signal of the ultra-wide band wireless sensors can be written as:

(19) $$\begin{array}{cc} r_{\mathrm{total}}\left(t\right) & =s^{U}\left(t\right)+s^{N}\left(t\right)+\sigma_{n}^{2}n\left(t\right). \end{array}$$

One of the key advantages of UWB signals is immunity to fading; the Rake combining receiver can exploit the high degree of diversity that results from a large number of multipath components (MPCs) [51]. Combining all resolvable paths as in the all-Rake (ARake) receiver provides the optimal performance. However, the number of MPCs that can be utilized in a typical Rake combiner is limited by power consumption constraints, complexity considerations and the availability of channel estimates. To reduce the computation complexity, selective Rake (SRake) and partial Rake (PRake) are also widely utilized. In this subsection, we consider a Rake receiver to combine a set of multipath components of the hybrid signal. Without loss of generality, we assume User 1 is the primary user of interest. To simplify the discussion, only symbol synchronization and the chip synchronization case are considered in this work. The local template signal for the *k*-th information bit can be expressed as:

(20) $$\begin{array}{c} s_{\mathrm{temp},[1]}\left(t\right)=\;\;\;\sum_{i=kN_{f}}^{\left(k+1\right)N_{f}-1}\;\;\;d_{\left[1\right]}\left(i\right)v_{\left[q\right]}\;\left(t-iT_{f}-c_{\left[1\right]}^{\mathrm{TH}}\left(i\right)T_{c}\right), \end{array}$$

where:

(21) $$\begin{array}{c} v_{\left[q\right]}\left(t\right)=\sum_{l_{r}=1}^{L_{r}}\psi_{l_{r}}p_{\mathrm{rx}}\left(t-\tau_{l_{r},\left[q\right]}\right), \end{array}$$

with $\Psi =[\psi_{1},\psi_{2},...,\psi_{L_{r}}]$ being the Rake combining weight coefficients [48,54,55,56]. The local template described by (20) and (21) can represent different multipath diversity combining schemes through assigning a proper value for the weight coefficients vector Ψ. Commonly, the fingers number $L_{r}$ of a practical Rake receiver should be smaller than the number of resolvable multipath $L_{p}$, with $L_{r}\leq L_{p}$. In an $L_{r}$ finger Rake, the weight coefficients for $L_{p}-L_{r}$ multipath components not used in the Rake receiver are set to zero while the remaining $L_{r}$ weight coefficients are determined according to a combining scheme, typically including maximal ratio combining (MRC) [55], equal gain combining (EGC) [51] and selection combining (SC) [54].

The output of the Rake receiver is given by:

(22) $$\begin{array}{c} y_{r}=\int \left\{s^{U}\left(t\right)+s^{N}\left(t\right)+\sigma_{n}^{2}n\left(t\right)\right\}s_{\mathrm{temp},[1]}\left(t\right)dt=U_{\mathrm{des}}+I_{\mathrm{mai}}+I_{\mathrm{nbi}}+N, \end{array}$$

where the first term $U_{\mathrm{des}}$ is due to the desired ultra-wide band signal:

(23) $$\begin{array}{c} U_{\mathrm{des}}=\sqrt{\frac{E_{1}}{N_{f}}}\Xi_{\left[1\right]}R_{u_{\left[q\right]}v_{\left[q\right]}}\left(0\right), \end{array}$$

with $R_{u_{\left[q\right]}v_{\left[q\right]}}\left(x\right)=\int u_{\left[q\right]}\left(t-x\right)v_{\left[q\right]}\left(x\right)dt$ denoting the cross-correlation between $u_{\left[q\right]}\left(t\right)$ of (16) and $v_{\left[q\right]}\left(t\right)$ of (21), $I_{\mathrm{mai}}$ is the MAI, $I_{\mathrm{nbi}}$ is the interference term caused by the narrow band interferences and *N* is the output noise, which is approximate to a normal distribution $N∼N(0,N_{f}\sigma_{n}^{2}E_{v_{\left[1\right]}})$ with $E_{v_{\left[1\right]}}=\int {\left(v_{\left[1\right]}\left(t\right)\right)}^{2}dt$.

Thus, the average probability of bit error can be expressed approximately as:

(24) $$\begin{array}{c} P_{e}\approx Q\left(\frac{\sqrt{E_{1}}R_{u_{\left[1\right]}v_{\left[1\right]}}\left(0\right)}{\sqrt{\frac{1}{N}\sum_{q=2}^{N_{u}}E_{\left[q\right]}\sigma_{\mathrm{mai},[q]}^{2}+\sigma_{\mathrm{nbi}}^{2}E_{v_{\left[1\right]}}+\sigma_{n}^{2}E_{v_{\left[1\right]}}}}\right). \end{array}$$

Usually, the received hybrid wireless signal is subject to the aggregation of the original UWB radio suffering from multi-path propagation, multiple access interferences, narrow band interferences from the pre-existing wireless communication system and additive white Gaussian noise. If one only considers the AWGN case, the optimum receiver could consist of a matched filter (correlation) receiver, where the hybrid received wireless signal is correlated with the transmitted ultra-wide band pulse $p_{tx}\left(t\right)$. Once multi-path propagation is considered, an ideal receiver will be replaced by the well-known Rake receiver [51] and channel equalizer [23], since the huge transmission bandwidths introduce a high degree of diversity at the receiver due to a large number of resolvable multi-path components. Commonly, the multiple access interferences (MAI) can be mitigated by designing and choosing the proper time-hopping sequences and direct spread spectrum codes [37,38] or utilizing multi-user detection (MUD) techniques [57]. In this paper, we focus on the in-band interferences from the other pre-existing narrow band wireless signals. However, the proposed method can be used combined with any other interference mitigation approaches and digital reception techniques.

## 4. NBI Suppression Algorithm

We should notice that the reception performance of ultra-wide band wireless sensors can be easily degraded by in-band pre-existing NBIs with high level power spectrum density. In practical application situation, in-band narrow band interferences (wireless systems, such as global systems for mobile (GSM) communications systems, wideband code-division multiple-access (WCDMA) mobile communication systems, time division-synchronous code division multiple access (TD-SCDMA) mobile communication systems, long-term evolution (LTE) mobile communication systems, universal mobile telecommunications (UMTS), satellite communication systems, global navigation satellite system (GNSS), Bluetooth, ZigBee, digital video broadcasting (DVB), wireless fidelity (WiFi) and RFID networks, etc.) is ubiquitous in the ultra-wide band radio devices, which makes the inherent in-band NBI immunity not sufficient enough. The performance of practical UWB wireless sensors will deteriorate, if no proper interference suppression countermeasure is employed in front of the receiver.

### 4.1. Single Narrow Band Interference Suppression

To simplify the discussion, without loss generality, we observe the one order complex-coefficient adaptive notch filter unit with a single constrained zero-pole pair first [35]. The transfer function of the one order complex-coefficient infinite impulse response (IIR) adaptive notch filter unit can be expressed as:

(25) $$\begin{array}{c} H_{1}\left(z\right)=\frac{z-z_{1}}{z-p_{1}}, \end{array}$$

where $z_{1}$ and $p_{1}$ denote the zero point and the pole point of the basic one order complex-coefficient adaptive notch filter unit, respectively. For the one order IIR notch filter, the stability condition requires that $p_{1}$ must be less than one.

**Figure 1:**
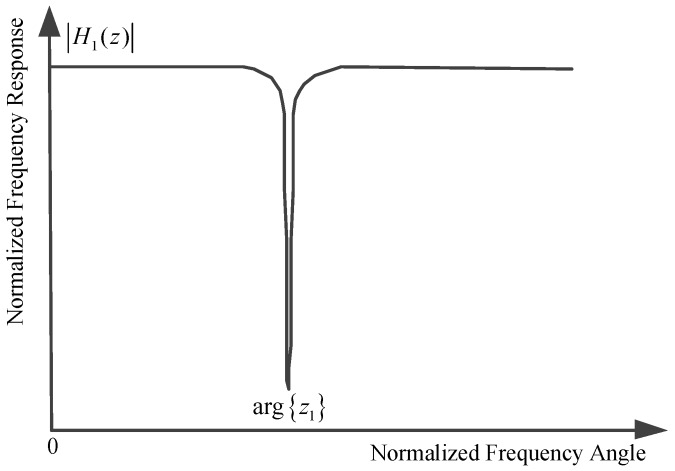
Normalized frequency response of one order complex-coefficient adaptive notch filter unit.

We constrain the zero-pole pair as follows: (1) the zero point is strictly located on the unit circle; (2) the pole point is located inside and close to the unit circle; (3) the zero point and the pole point are under the same frequency angle related to the central frequency of narrow band interference. In other words, the relationship between the zero point and the pole point can be described as:

(26) $$\begin{array}{c} p_{1}=\rho z_{1}=\rho \exp\left(j\chi_{1}\right), \end{array}$$

where $\chi_{1}$ denotes the notch angular frequency with $\chi_{1}=arg\left\{z_{1}\right\}$, $0\leq \chi_{1}<2\pi$ and ρ represents the distance between the origin point and the pole point in the complex plane with 0<ρ<1. As depicted in Figure 1, the zero point on the unit circle brings about an infinite depth notch around $\chi_{1}$, and the parameter ρ controls the bandwidth of the notch. We should note that the parameter ρ is chosen to be close to, but less than one to obtain the narrow band notch and avoid any filter instability problems. In Figure 1, the mathematic mark arg$\left\{z_{1}\right\}$ denotes taking the argument of the complex variable $z_{1}$. From (25) and (26), we can obtain the following relationship in the time domain:

(27) $$\begin{array}{c} y_{1}\left(n\right)-\rho h_{1}\left(n\right)y_{1}\left(n-1\right)=x_{1}\left(n\right)-h_{1}\left(n\right)x_{1}\left(n-1\right), \end{array}$$

where $x_{1}\left(n\right)$ (n=1,2,3...) denotes the hybrid input of the one order complex-coefficient ANF unit with a single zero-pole pair including the useful ultra-wide band wireless signal, in-band pre-existing strong NBIs, MAI and additive white Gaussian noises, and $h_{1}\left(n\right)$ is the adaptive complex-coefficient of the basic one order adaptive notch filter unit. Due to the above-mentioned constrained relationship, the coefficient of notch filter can be reexpressed as:

(28) $$\begin{array}{c} h_{1}\left(n\right)=|h_{1}\left(n\right)|\exp\left\{j\arg\left\{h_{1}\left(n\right)\right\}\right\}=\exp\left\{j\chi_{1}\left(n\right)\right\}, \end{array}$$

where $\chi_{1}\left(n\right)$ denotes the instantaneous phase relating to angular frequency for the *n*-th moment.

**Figure 2:**
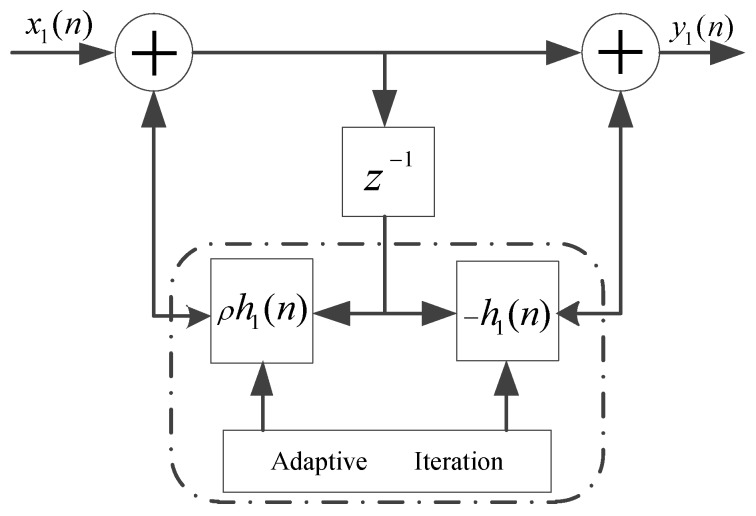
Implementation of the one order complex-coefficient notch filter unit with a single constrained zero-pole pair.

The detailed implementation of the one order complex-coefficient adaptive notch filter unit with a single constrained zero-pole pair is depicted in Figure 2. Once the frequency angle $\chi_{1}\left(n\right)$ has converged to the objective central frequency of NBI, the adaptive notch filter unit will cut off the corresponding in-band strong NBI component. In this place, we define the error function $e_{1}\left(n\right)$ as:

(29) $$\begin{array}{c} e_{1}\left(n\right)=x_{1}\left(n\right)-h_{1}\left(n\right)x_{1}\left(n-1\right)+\rho h_{1}\left(n\right)y_{1}\left(n-1\right). \end{array}$$

According to adaptive filter theory, the objection is to minimize the mean square error (MSE) of $e_{1}\left(n\right)$; hence, the cost function becomes:

(30) $$\begin{array}{c} C_{1}\left(n\right)=E\left\{|e_{1}\left(n\right){|}^{2}\right\}\approx \frac{1}{N}\sum_{n=1}^{N}\left\{e_{1}\left(n\right)e_{1}^{*}\left(n\right)\right\}, \end{array}$$

where * denotes the complex conjugate.

The adaptive weight coefficient of the one-order adaptive notch filter with a single zero-pole pair is determined by minimizing the cost function $C_{1}\left(n\right)$. Thus, the iteration relationship can be considered as the gradient of $C_{1}\left(n\right)$ with the instantaneous angular frequency $\chi_{1}\left(n\right)$, which can be given by:

(31) $$\begin{array}{cc} \frac{\nabla C_{1}\left(n\right)}{\nabla \chi_{1}\left(n\right)}= & jx_{1}\left(n\right)x_{1}^{*}\left(n-1\right)\exp\left\{-j\chi_{1}\left(n\right)\right\}-jx_{1}^{*}\left(n\right)x_{1}\left(n-1\right)\exp\left\{j\chi_{1}\left(n\right)\right\}\\ & +j\rho x_{1}^{*}\left(n\right)y_{1}\left(n-1\right)\exp\left\{j\chi_{1}\left(n\right)\right\}-j\rho x_{1}\left(n\right)y_{1}^{*}\left(n-1\right)\exp\left\{-j\chi_{1}\left(n\right)\right\}. \end{array}$$

We denote $T_{1}\left(n\right)\;=\;x_{1}\left(n\right)x_{1}^{*}\left(n-1\right)\exp\left\{-j\chi_{1}\left(n\right)\right\}+\rho x_{1}^{*}\left(n\right)y_{1}\left(n-1\right)\exp\left\{j\chi_{1}\left(n\right)\right\}$; thus, the following one-step adaptive frequency angle iteration relationship can be obtained:

(32) $$\begin{array}{c} \chi_{1}\left(n\;+\;1\right)=\chi_{1}\left(n\right)+\mu \mathrm{Imag}\left[T_{1}\left(n\right)\right], \end{array}$$

where μ denotes the adaptive step-size associated with the convergence rate of the proposed ANF and Imag denotes taking the imaginary part of the complex variabled.

From (32), the following iteration relationship can be obtained:

(33) $$\begin{array}{c} \exp\left\{j\chi_{1}\left(n\;+\;1\right)\right\}=\exp\left\{j\chi_{1}\left(n\right)\right\}\cdot \exp\left\{j\mu \mathrm{Imag}\left[T_{1}\left(n\right)\right]\right\}. \end{array}$$

Expending (33) with the second order Taylor series expansion yields:

(34) $$\begin{array}{c} \exp\left\{j\chi_{1}\left(n\;+\;1\right)\right\}\;=\;\exp\left\{j\chi_{1}\left(n\right)\right\}\left\{1\;+\;j\mu \mathrm{Imag}\left[T_{1}\left(n\right)\right]\;+\frac{1}{2!}{\left\{j\mu \mathrm{Imag}\left[T_{1}\left(n\right)\right]\right\}}^{2}\;\;+\;o{\left\{j\mu \mathrm{Imag}\left[T_{1}\left(n\right)\right]\right\}}^{2}\right\}, \end{array}$$

where a function f(x) is o(g(x)) if $lim_{x\to 0}f\left(x\right)/g\left(x\right)=0$. Substituting $h_{1}\left(n\right)=\exp\left\{j\chi_{1}\left(n\right)\right\}$ into (34), we obtain the iteration relationship of adaptive weight coefficient as:

(35) $$\begin{array}{c} \;h_{1}\left(n\;+\;1\right)=h_{1}\left(n\right)\left\{1+j\mu \mathrm{Imag}[T_{1}\left(n\right)]-\frac{1}{2}{\left\{\mu \mathrm{Imag}\left[T_{1}\left(n\right)\right]\right\}}^{2}\right\}. \end{array}$$

To clarify the proposed adaptive weight coefficient iteration algorithm, we summarize the procedure of the above-mentioned complex-coefficient ANF unit with a single zero-pole pair in Table 1.

### 4.2. Linear Cascaded High Order Complex-Coefficient Adaptive Notch Filter

For the multiple in-band strong NBI case, it is inevitable to utilize high-order ANF. If we directly expand the numerator polynomial into the high-order finite impulse response (FIR) filter and adjust the denominator polynomial at the same time, it is rather hard to guarantee all of the pole points distribute inside the unit circle to satisfy stability.

**Figure 3:**
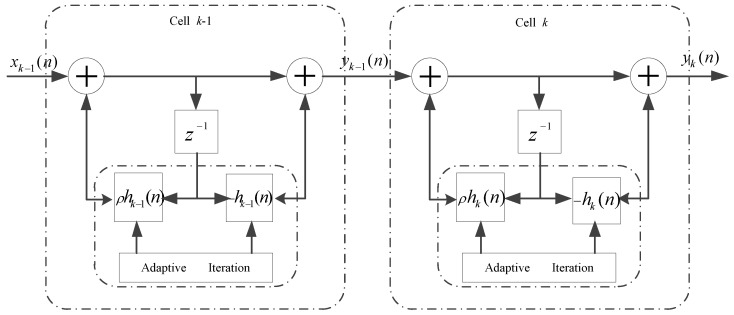
Implementation of linear cascaded complex-coefficient high order adaptive notch filter.

A feasible approach is to cascade the proposed one order complex efficient IIR ANF units into linear cascaded (LC) high order ANF [36]. The implementation of the linear cascaded high order complex-coefficient ANF is depicted in Figure 3. The transfer function of *K*-order linear cascaded complex efficient ANF becomes:

(36) $$\begin{array}{c} H\left(z\right)=\prod_{k=1}^{K}H_{k}\left(z\right)=\prod_{k=1}^{K}\frac{1-h_{k}z^{-1}}{1-\rho h_{k}z^{-1}}, \end{array}$$

where $H_{k}\left(z\right)$ denotes the transfer function of the *k*-th basic IIR notch filter unit and $h_{k}$ denotes the *k*-th adaptive weight coefficient. The frequency response of the above transfer function has a unified gain at all frequencies; except at the notch frequencies in the present of narrow band interferences, their gains approximate to zero.

In the LC high order complex-coefficient ANF framework, the output of the (k−1)-th one order ANF unit can be considered as the input of the *k*-th complex-coefficient one order ANF unit, so the difference equation of the *k*-th section is given by:

(37) $$\begin{array}{c} y_{k}\left(n\right)=y_{k-1}\left(n\right)-h_{k}\left(n\right)y_{k-1}\left(n-1\right)+\rho h_{k}\left(n\right)y_{k}\left(n-1\right). \end{array}$$

Thus, the intermediate variable of the *k*-th one order complex-coefficient ANF unit becomes:

(38) $$\begin{array}{c} T_{k}\left(n\right)=y_{k-1}\left(n\right)y_{k-1}^{*}\left(n-1\right)h_{k}^{*}\left(n\right)+\rho y_{k-1}^{*}\left(n\right)y_{k}\left(n-1\right)h_{k}\left(n\right). \end{array}$$

The complex coefficients’ iteration relationship for the *k*-th one order complex-coefficient IIR ANF unit can be expressed as:

(39) $$\begin{array}{c} \;h_{k}\left(n\;+\;1\right)=h_{k}\left(n\right)\left\{1+j\mu \mathrm{Imag}[T_{k}\left(n\right)]-\frac{1}{2}{\left\{\mu \mathrm{Imag}\left[T_{k}\left(n\right)\right]\right\}}^{2}\right\}. \end{array}$$

In the LC *K*-order complex-coefficient ANF, the adaptive iteration relationship of each one order ANF unit can be updated separately, since the objection frequency of NBI in each ANF unit is different. The processing of the LC high order complex-coefficient ANF algorithm based on one order ANF unit can be found in Table 2.

### 4.3. Cyclic Cascaded High Order Complex-Coefficient Matrix Adaptive Notch Filter

**Figure 4:**
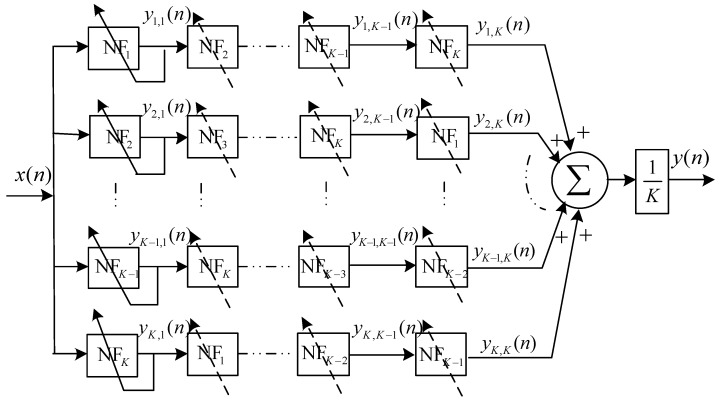
The framework of cyclic cascaded high order complex-coefficient matrix adaptive notch filter based on one order complex-coefficient adaptive notch filter units.

In the linear cascaded high order complex-coefficient adaptive notch filter, the tracking performance will degrade, since the input of the current section of the adaptive notch filter unit is the output of the previous one. Moreover, the convergence rate will decrease. To overcome the above shortcomings, we propose a cyclic cascaded framework, which is depicted in Figure 4. A *K*-order cyclic cascaded matrix adaptive notch filter is composed of the K×K basic one order IIR notch filter units. Apparently, K×K adaptive coefficients should be calculated for each iteration in a *K*-order cyclic cascaded high order matrix adaptive notch filter. In fact, only *K* active adaptive weight coefficients in the left column as depicted under the solid line in Figure 4 should be determined according to the weight coefficient iteration algorithm. Meanwhile, the updated complex value weight coefficients are copied to the other corresponding adaptive notch filter units at each iteration, which reduced the algorithm complexity greatly. For a *k* order CCHOMANF, only 12k multipliers and 5k adders are used to fulfil the update of the adaptive weight coefficients. Commonly, the number of in-band narrow band interferences with different central frequencies is not more than 10 in practical ultra-wide band wireless sensor networks. In other words, the order of CCHOMANF is not very large, so we can set 3≤k<10, which makes the computation complexity of the proposed narrow band interference suppression algorithm reasonable.

To simplify the discussion, we define adaptive notch filter cell NF${}_{k}$ (k=1,2,...,K) as the *k*-th active basic adaptive notch filter unit, which is depicted in left column in Figure 4. The transfer function of the *k*-th active adaptive notch filter is given by:

(40) $$\begin{array}{c} H_{k}\left(z\right)=\frac{1-h_{k}z^{-1}}{1-\rho h_{k}z^{-1}}. \end{array}$$

Hence, the transfer function of cyclic cascaded high order matrix adaptive notch filter can be described as:

(41) $$\begin{array}{c} H\left(z\right)=\frac{1}{K}\sum_{m=1}^{K}\prod_{k=1}^{K}H_{k}\left(z\right), \end{array}$$

where $H_{k}\left(z\right)$ designates the *k*-th one order complex-coefficient IIR adaptive notch filter unit.

**Figure 5:**
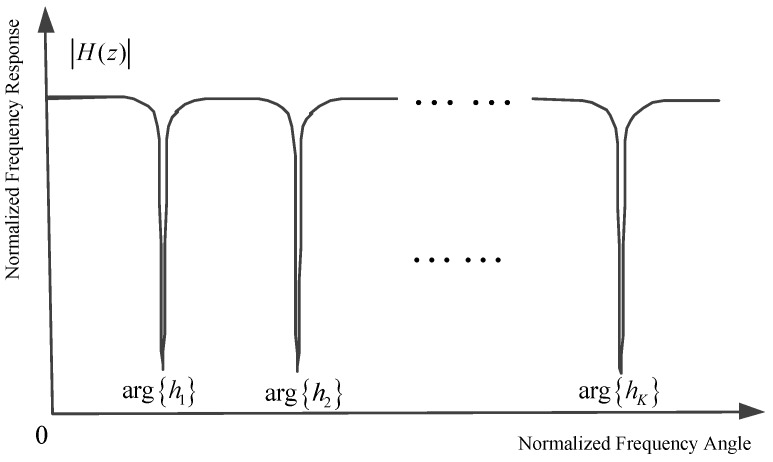
Normalized frequency response of the *K* order complex-coefficient cyclic cascaded matrix adaptive notch filter.

The normalized frequency response of *K* order CCHOMANF is depicted in Figure 5. In theory, a *K* order CCHOMANF can suppress *K* narrow band interferences. Substantially, in the cyclic cascaded high order matrix adaptive notch filter, the entire complex plane is divided into *K* circulator sections with each section being monitored by a basic one-order adaptive notch filter unit. The specific number of cascaded units is dependent on the practical application situation. In this place, we note that the order number *K* of CCHOMANF does not need to be adjusted in different application scenarios, even if the number of narrow band interferences is variable. Normally, we consider 5≤K≤10 (let the order number *K* be lager than the number of narrow band interferences in different frequencies). When *K* is more than the number of narrow band interferences $N_{\mathrm{NBIs}}$, $K\;-\;N_{\mathrm{NBIs}}$ redundant adaptive notch filter units will present. The redundant complex-coefficient adaptive notch filter cells will vibrate around the central frequencies of narrow band interferences close to them. Therefore, the proposed CCHOMANF still runs soundly, even though the order of the CCHOMANF *K* is not equal to the number of narrow band interferences. It is worth noting that, in the initialization stage, we set $h_{k}\left(1\right)$ as follows: $h_{1}\left(1\right)=1,h_{2}\left(1\right)=cos\left(2\pi /K\right)+jsin\left(2\pi /K\right),h_{k}\left(1\right)=cos\left(2\left(k-1\right)\pi /K\right)+jsin\left(2\left(k-1\right)\pi /K\right)$, which guarantees that different adaptive notch filter units converge to different narrow band interferences close to them, rather than some certain narrow band interference.

Through the aforementioned construction, the cyclic cascaded high order adaptive notch filter has the following excellent properties: (1) all of the weight coefficients are sought simultaneously as depicted in the left column in Figure 4, alleviating the associated polynomial deflation; (2) a priori information or constraints on the zero-pole locations are easily incorporated into the estimation procedure; (3) the iterations utilizing the gradient-descent algorithm have been proven to be relatively effective with a minimum computational complexity; and (4) the imperfect knowledge of the practical ultra-wide band channel has tiny effects on the proposed narrow band interferences suppression since the interference components will be cleaned off rather than be recovered.

## 5. Numerical Simulation Results and Reception Performance Analysis

In this section, the numerical simulation results are presented to illustrate the reception performance of time hopping pulse position modulation impulse-based ultra-wide band wireless sensors and the performance enhancement provided by the complex-coefficient adaptive notch filter narrow band interference suppression algorithm developed in this paper under different channel propagation environments. We consider a second derivative of the Gaussian monocycle transmitted impulse waveform with $\tau_{p}=0.0625$ ns. The frame length $T_{f}=512$ ns; the duration of the chip $T_{c}=0.25$ ns; the number of time slots per frame is 2048; the number of transmitted base band pulses per symbol $N_{s}=4$; and the number of active users $N_{u}=8$. We suppose the time hopping codes for different users are orthogonal.

**Figure 6:**
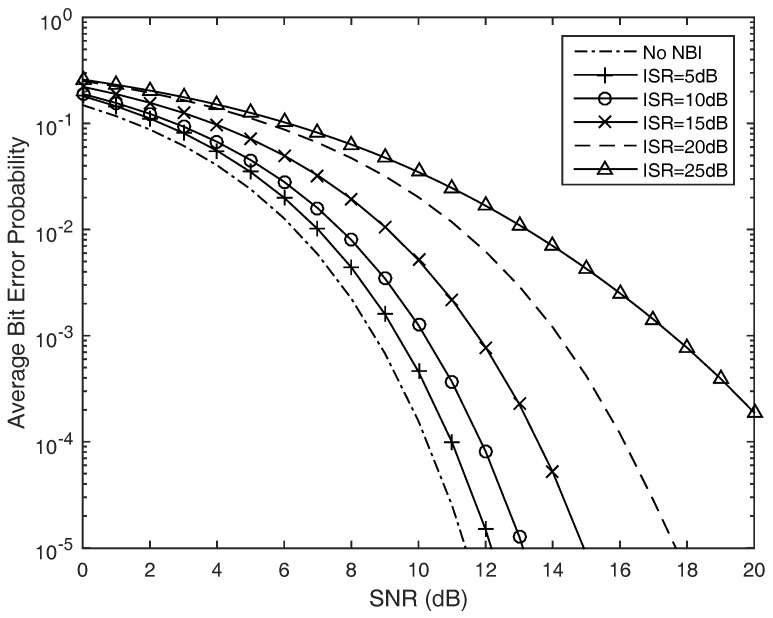
Average bit error probability of the TH-pulse position modulation (PPM) UWB wireless sensors in the unfaded scenario with the narrow band interferences under different power levels.

We first observe the reception performance of the time hopping pulse position modulation impulse ultra-wide band signal in the present of narrow band interferences under additive white Gaussian noise (unfaded scenario) channel. Figure 6 shows the simulated average bit error probability (BEP) versus average signal to noise ratio (SNR) for different values of the narrow band interference to signal ratio (ISR). We can find that the narrow band interference will deteriorate the system performance, especially when the ISR is more than 10 dB.

**Figure 7:**
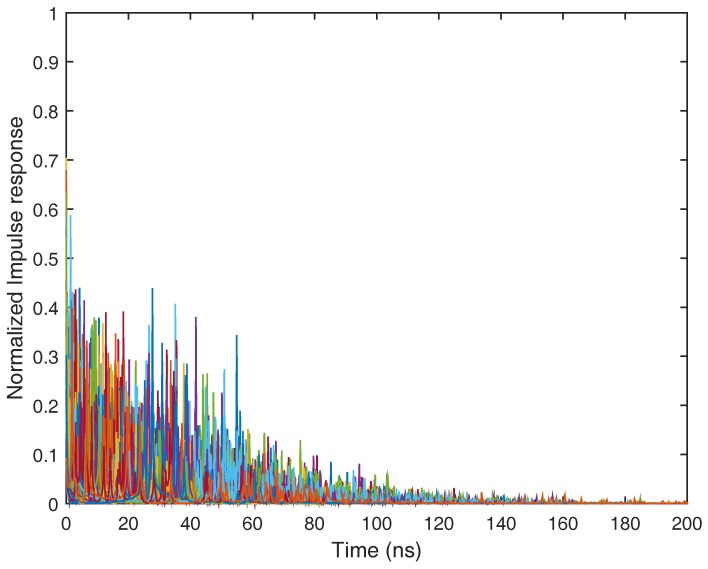
Normalized impulse response of the IEEE 802.15.4a CM1 channel model for the indoor residential line of sight scenario.

The practical ultra-wide band radio propagation environment is a dense multipath fading channel. A number of UWB channel models have been proposed in the last decade: [58] suggested a model for the frequency range below 1 GHz. The IEEE 802.15.3a group developed a channel model that is valid from 3 GHz to 10 GHz, but is designed only for indoor residential and office environments, and the distance between transmitter and receiver is restricted to 10 m [59]. The IEEE 802.15.3a channel model is parameterized for line of sight (LOS), as well as non-line of sight (NLOS) circumstances in residential, industrial, office, farm and open outdoor environments. Based on more practical measurements than the IEEE 802.15.3a channel model, the IEEE 802.15.4a work groups provided a more general parameterized ultra-wide band channel model from 2 GHz to 10 GHz. The standard ultra-wide band channel model also covers office, farm, residential, industrial, open outdoor environments and body-area networks with both LOS and NLOS cases [50,60]. In this place, we first consider the IEEE 802.15.4a CM1 channel model for the indoor residential environment under the line of sight case. The normalized impulse response in the IEEE 802.15.4a CM1 channel model is depicted in Figure 7. The impulse response of the ultra-wide band CM1 channel consists of hundreds of resolvable multi-path components. It is not difficult to notice that the channel impulse response of CM1 is the sum of attenuated, delayed and distorted multipath components, and in this case, well-known Rake receivers can be used to collect the dispersive energy of multipath components. A Rake receiver can consist of a matched filter with a tapped delay line matching the impulse response of the practical ultra-wide band channel. It also can be replaced by a set of time delay correlators that are sampled at the delays related to specific multipath components.

**Figure 8:**
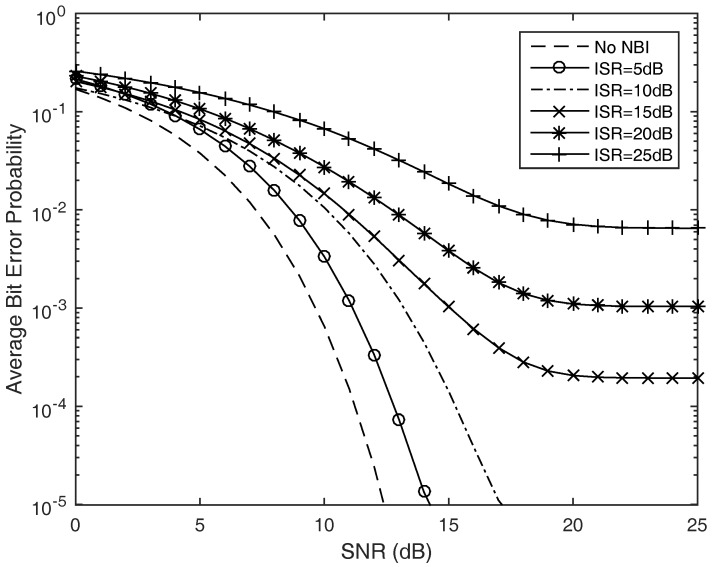
Average bit error probability for the TH-PPM UWB wireless sensors with narrow band interferences under the IEEE 802.15.4a CM1 multipath fading channel.

We next consider the case in which the time hopping ultra-wide band wireless signal undergoes multipath fading. More specifically, the IEEE 802.15.4a CM1 channel model for the indoor residential environment under line of sight case is considered. In the simulations, selective Rake combining is employed, where the number of fingers $L_{r}$ is chosen big enough. The results are averaged over 1000 channel realizations, each associated with a random TH code. It can be seen from Figure 8 that the performance is severely degraded by the presence of fading on the desired signal comparing the average bit error probability with the previous scenarios. When the ISR is more than 20 dB, the system performance is unsatisfactory if no proper narrow band interference suppression is employed. The proposed interference suppression algorithm can greatly improve the reception performance under the strong in-band interferences case.

**Figure 9:**
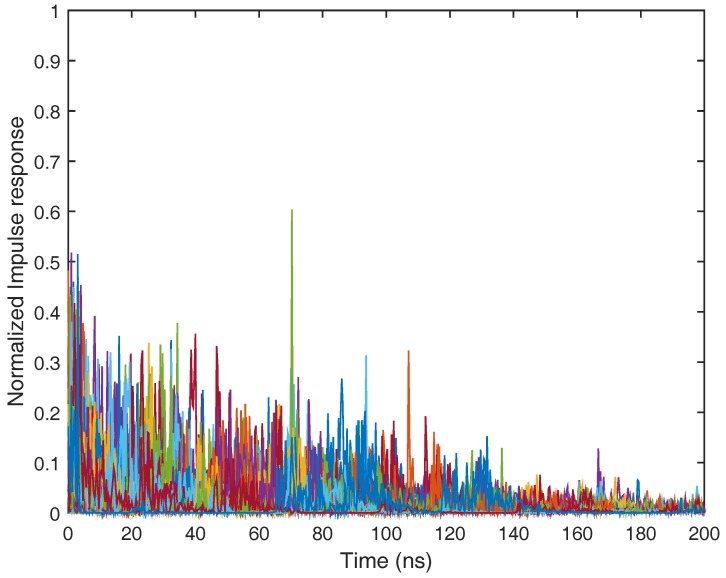
Normalized impulse response of the IEEE 802.15.4a CM5 channel model for the outdoor line of sight scenario.

**Figure 10:**
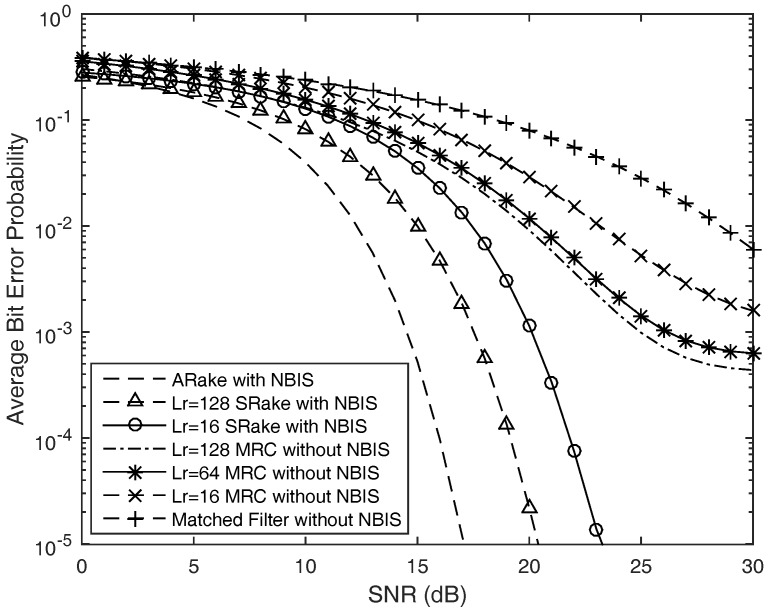
Average bit error probability versus SNR of the time hopping ultra-wide band wireless sensors receiver considered with and without the proposed narrow band interference (NBI) scheme.

Finally, to validate the practical amount of performance enhancement obtained by the complex-coefficient NBIs scheme proposed in this article, we investigate the average bit error probability of time hopping code division pulse position modulation ultra-wide band wireless reception in the present of multiple narrow band interferences with and without the designed complex-coefficient adaptive notch filters. The simulation is executed under IEEE 802.15.4a CM5 channel model for the outdoor line of sight scenario. The normalized impulse response of IEEE 802.15.4a CM5 channel model is depicted in Figure 9. Compared with the simulation in Figure 7, we can find that the energy of the multipath component is more dispersive in the outdoor environment. The reception performance under the IEEE802.15.4a CM5 channel model is depicted in Figure 10. In this scenario, the UWB wireless sensors suffered from four in-band strong narrow band interferences with four different central frequencies. In the simulation, we suppose that the signal to interference ratio is −30 dB. From the numerical simulation results, we can notice that the reception performance is worst when the receiver consists of the matched filter without the proposed narrow band interferences’ suppression; the average bit error probability is larger than $10^{-2}$ even if the average signal to noise ratio is more than 25 dB through this kind of reception. The performance of Rake combining outperforms the matched filter scheme, and the A-Rake scheme with the proposed NBIs can obtain the best reception performance. In addition, we can find that the reception performance of SRake with NBIs outperforms the case of MRC combining without the proposed NBIs since the proposed NBI algorithm can improve the reception SINR. Both the proposed NBI approach and Rake combining receiver can elevate the reception performance of ultra-wide band wireless sensors, since the complex-coefficient adaptive notch filtering algorithm proposed in this article can remove the strong NBIs with slight distortion and the Rake combining receiver will collect the dispersed energy of the multi-path components due to the large number of resolvable multipath components in cognitive ultra-wide band wireless sensor networks.

## 6. Conclusions

Impulse-based time hopping ultra-wide band radios are particularly suitable for underlying low-cost low-power consumption communications and positioning, over a huge frequency band where, possibly, other legacy narrow band and broadband wireless systems and electronic devices are active. Both the interferences coming from the narrow band users and the interferences coming from ultra-wide band users should be considered. The FCC’s frequency spectral and power spectral density regulations have set restricted conditions that limit the interferences from ultra-wide band radiators to other pre-existing wireless communication systems and electronic devices; however, the issue for in-band strong narrow band interferences suppression in ultra-wide band wireless sensor networks is left to the ingenuity of the ultra-wide band wireless sensors designer. In this article, we develop a novel one order complex-coefficient adaptive notch filter with a single zero-pole pair for in-band interferences suppression in cognitive ultra-wide band wireless sensor networks. The proposed algorithm only requires a single adaptive weight coefficient to notch out one narrow band interference. To cope with multiple in-band narrow band interferences simultaneously, a linear cascaded high order adaptive interference suppression and a novel cyclic cascaded high order matrix adaptive interference suppression scheme are also presented. The theoretical analysis and numerical simulation experimentation results indicate that the interference tolerance margin of time hopping based ultra-wide band wireless sensor networks can be greatly enhanced by the proposed complex-coefficient cyclic cascaded high order matrix adaptive filters, which have excellent suppression effects to in-band strong narrow band interferences in low-cost low-power-consumption cognitive ultra-wide band sensor networks with a substantial SINR promotion acquired. In addition, the proposed complex-coefficient adaptive in-band interference suppression processing can be executed before the demodulation module; it is also suitable for front end in-band interference elimination of the non-coherent impulse-based ultra-wide band wireless sensor networks connected with Rake receiver and multiuser detector.

## Figures and Tables

**Table 1:** The detailed procedure of the one order complex-coefficient notch filter unit with a single constrained zero-pole pair.

Step 1	initialize system variables $x_{1}\left(0\right)=0.1$, $y_{1}\left(0\right)=0.1$, set initial value $h_{1}\left(1\right)=1$;
Step 2	for n=1,2,3..., calculate the intermediate variables according to $T_{1}\left(n\right)=x_{1}\left(n\right)x_{1}^{*}\left(n-1\right)h_{1}^{*}\left(n\right)+\rho x_{1}^{*}\left(n\right)y_{1}\left(n-1\right)h_{1}\left(n\right)$;
Step 3	for n=1,2,3..., calculate the complex coefficient according to $h_{1}\left(n\;+\;1\right)\;=\;h_{1}\left(n\right)\left\{1\;+j\mu \mathrm{Imag}[T_{1}\left(n\right)]\;-\;\frac{1}{2}{\left\{\mu \mathrm{Imag}\left[T_{1}\left(n\right)\right]\right\}}^{2}\right\}$;
Step 4	calculate the output of the adaptive notch filter unit according to $y_{1}\left(n\right)=x_{1}\left(n\right)-h_{1}\left(n\right)x_{1}\left(n-1\right)+\rho h_{1}\left(n\right)y_{1}\left(n-1\right)$, and then turn back to Step 2.

**Table 2:** The procedure of the linear cascaded high order complex-coefficient adaptive notch filter algorithm based on the basic one order adaptive notch filter unit.

Step 1	initialize system variables $x_{1}\left(0\right)=0.1$, $y_{1}\left(0\right)=0.1$, $y_{k}\left(0\right)=0.1$, set initial value $h_{k}\left(1\right)=1$, for k=1,2...,K;
Step 2	for n=1,2,3..., calculate the intermediate variable according to $T_{k}\left(n\right)=y_{k-1}\left(n\right)y_{k-1}^{*}\left(n-1\right)h_{k}^{*}\left(n\right)+\rho y_{k-1}^{*}\left(n\right)y_{k}\left(n-1\right)h_{k}\left(n\right)$;
Step 3	for n=1,2,3..., calculate the adaptive instantaneous complex weight coefficients according to $h_{k}\left(n\;+\;1\right)\;=\;h_{k}\left(n\right)\left\{1\;+j\mu \mathrm{Imag}[T_{k}\left(n\right)]\;-\;\frac{1}{2}{\left\{\mu \mathrm{Imag}\left[T_{k}\left(n\right)\right]\right\}}^{2}\right\}$;
Step 4	calculate the output of the adaptive notch filter unit according to $y_{k}\left(n\right)=y_{k-1}\left(n\right)-h_{k}\left(n\right)y_{k-1}\left(n-1\right)+\rho h_{k}\left(n\right)y_{k}\left(n-1\right)$ and then turn back to Step 2.
